# The C-Terminal Domain of the MutL Homolog from *Neisseria gonorrhoeae* Forms an Inverted Homodimer

**DOI:** 10.1371/journal.pone.0013726

**Published:** 2010-10-28

**Authors:** Sivakumar Namadurai, Deepti Jain, Dhananjay S. Kulkarni, Chaitanya R. Tabib, Peter Friedhoff, Desirazu N. Rao, Deepak T. Nair

**Affiliations:** 1 Laboratory 4, National Centre for Biological Sciences, Bangalore, India; 2 Department of Biochemistry, Indian Institute of Science, Bangalore, India; 3 Institut für Biochemie (FB 08), Justus-Liebig-Universität, Giessen, Germany; University of Hyderabad, India

## Abstract

The mismatch repair (MMR) pathway serves to maintain the integrity of the genome by removing mispaired bases from the newly synthesized strand. In *E. coli*, MutS, MutL and MutH coordinate to discriminate the daughter strand through a mechanism involving lack of methylation on the new strand. This facilitates the creation of a nick by MutH in the daughter strand to initiate mismatch repair. Many bacteria and eukaryotes, including humans, do not possess a homolog of MutH. Although the exact strategy for strand discrimination in these organisms is yet to be ascertained, the required nicking endonuclease activity is resident in the C-terminal domain of MutL. This activity is dependent on the integrity of a conserved metal binding motif. Unlike their eukaryotic counterparts, MutL in bacteria like *Neisseria* exist in the form of a homodimer. Even though this homodimer would possess two active sites, it still acts a nicking endonuclease. Here, we present the crystal structure of the C-terminal domain (CTD) of the MutL homolog of *Neisseria gonorrhoeae* (NgoL) determined to a resolution of 2.4 Å. The structure shows that the metal binding motif exists in a helical configuration and that four of the six conserved motifs in the MutL family, including the metal binding site, localize together to form a composite active site. NgoL-CTD exists in the form of an elongated inverted homodimer stabilized by a hydrophobic interface rich in leucines. The inverted arrangement places the two composite active sites in each subunit on opposite lateral sides of the homodimer. Such an arrangement raises the possibility that one of the active sites is occluded due to interaction of NgoL with other protein factors involved in MMR. The presentation of only one active site to substrate DNA will ensure that nicking of only one strand occurs to prevent inadvertent and deleterious double stranded cleavage.

## Introduction

The blueprint of life for each organism is resident in genomic DNA. Generally, for all cellular processes to function properly, the integrity of the genome has to be maintained. Even though the molecules and processes involved in replicating the genome function very efficiently to reduce the number of mutations, it has been observed that errors still arise during genome duplication [1]. In addition, mismatches also arise during homologous recombination and the deamination of 5-methylcytosine to thymine [1]. These mismatches will give rise to mutations after a round of replication that can be deleterious or silent. Additionally, a single mismatch in the genome is enough to hinder critical processes such as recombination and transcription [2], [3]. Due to the deleterious effects of mismatches there is a need for a system that will correct the errors that escape detection by the proofreading activity of DNA Polymerases. The specific and general molecules associated with the mismatch repair (MMR) pathway fulfill this requirement [4].

The MMR system serves to maintain the integrity of the genome by removing any errors- in the form of non-Watson-Crick base pairs- that appear during template-dependent DNA synthesis. To avoid creating mutations, the mismatches must be repaired according to the identity of the base in the parent strand. Therefore, it is extremely critical that the MMR system should have a strategy to identify the parent strand. In *E.coli*, this is accomplished by marking the parent strand through the action of Dam methylase. This enzyme methylates DNA at the N6 position of adenine within 5′-GATC-3′ sequences that are found interspersed throughout the genome. The presence of unmethylated adenine in the GATC sequences of the newly synthesized strand facilitates strand discrimination. The mismatch repair system in *E.coli* is composed of 11 protein components of which, MutS, MutL and MutH are specific to this pathway [4], [5]. The importance of this pathway can be understood from the fact that mutations in genes involved in MMR in humans have been implicated in the onset of hereditary non polyposis colon cancer (HNPCC) [6]. In *E. coli*, the primary step involves the recognition of the mismatch by MutS. This association is stabilized by the ATP-dependent recruitment of the protein MutL. MutH binds to hemi-methylated GATC sites but its latent endonuclease activity can only be activated through association with the MutL-MutS complex. Several models have been proposed for coupling mismatch recognition to strand discrimination [7]. In the sliding clamp model, it is believed that the MutL-MutS complex scans for a GATC site on either side of the mismatch by looping the DNA through a large central cavity present in MutL [8]. When the MutL-MutS complex encounters the protein MutH bound to the hemimethylated GATC sequences, the latent nicking endonucleolytic activity present in the latter is activated. A nick is created by MutH opposite the methylated adenine of the GATC site and this nick allows UvrD helicase to unwind double stranded DNA in the direction of the mismatch. This is followed by cleavage of the resulting single stranded region in the daughter strand by specific exonucleases with the correct directionality (5′ to 3′ or 3′ to 5′). The entire process results in the removal of a stretch of newly synthesized DNA containing the mismatch. The gap formed is filled in by high-fidelity DNA polymerase III and the nick is stitched by DNA ligase. Overall, the mismatch repair pathway in *E.coli* has been worked out in some detail at biochemical and structural levels [4], [5].

In case of eukaryotes and many bacteria, sequence analysis has failed to detect the presence of a MutH homolog. Many of these organisms also do not show the presence of a GATC methylase. Therefore, the strategy for strand discrimination as well as the mechanism for creation of a nick in the daughter strand to initiate excision will be different in case of these organisms. Although the exact mechanism for strand discrimination is yet to be ascertained, it has been shown in case of humans and yeast that the endonucleolytic activity is resident in the MutL homolog [9], [10], [11]. In case of humans, there exist three MutL homologs and they are present in the form of heterodimers. The three homologs MutLα, MutLβ and MutLγ are formed by the dimerization of MLH1 with PMS2, PMS1 and MLH3, respectively, The endonucleolytic activity necessary to create a nick is resident in the C-terminal domain of PMS2 and MLH3 and is dependent on the integrity of a conserved motif [DQHA(X)_2_E(X)_2_E) that was shown to be important for metal binding [9]. PMS1 does not have this motif and MutLβ does not appear to possess endonucleolytic activity. Overall, the protein responsible for creation of a nick in the absence of MutH to initiate mismatch repair has been identified. However, the chemical mechanism responsible for this activity remains elusive as does the strategy for strand discrimination. Very recently, studies on the human system revealed that the interaction between PCNA and MutLα can direct its nicking activity to one strand that contains already a preexisting nick [12].

Most bacteria, like the pathogen *Neisseria gonorrhoeae*, do not have homologs of MutH. The N-terminal domain (harboring the ATPase activity) of MutL homolog of this organism (NgoL) has high sequence similarity with that of the *E. coli* protein (EcoL). However the sequence of the C-terminal domain (known to aid dimerization) is significantly different from that of EcoL and harbors the metal binding motif leading to the hypothesis that the endonucleolytic activity might be resident in this domain. Duppatala and co-workers were able to show that this is indeed true and the integrity of the metal binding motif is critical for endonucleolytic activity [13]. The CTD alone was proficient in endonucleolytic activity and required the presence of Mg^2+^ or Mn^2+^ ions for this activity.

Unlike the eukaryotic MutL homologs, NgoL has been shown to exist as a homodimer in solution [13]. The fact that NgoL harbors a nicking endonuclease activity in its CTD presents a conundrum as to how a homodimeric molecule with two active sites does not create double-strand breaks. Additionally, the chemical mechanism utilized by the metal-binding motif for endonucleolytic activity remains to be ascertained. To answer these questions, we have determined the crystal structure of the C-terminal domain of NgoL (NgoL-CTD). The structure shows that although the order and length of secondary structural elements in the monomer is similar to EcoL, their relative spatial orientation is substantially different. Moreover, the structure provides a three dimensional view of the sequence motifs that have been shown to be conserved in the MutL family [14], [15]. During the preparation of this manuscript, the structure of the C-terminal domain of the MutL homolog from *Bacillus subtilis* (BsuL-CTD) was published and a comprehensive comparison between the structures of NgoL-CTD and BsuL-CTD (Zn^2+^ bound crystal form, PDB code:3KDK) is also presented [16]. It is seen that NgoL-CTD exists in the form of an inverted homodimer that is stabilized by extensive hydrophobic interactions. It is possible that such an arrangement allows for the presentation of only one active site to DNA and thus, provides a basis for the observed nicking activity.

## Results

### Structure Determination

**Figure 1:**
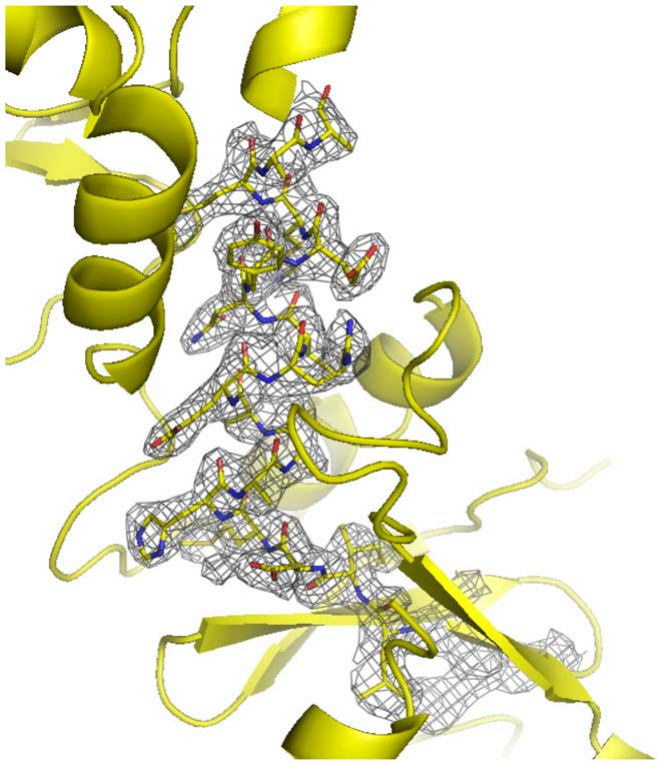
Electron Density Map. A section of a composite Omit Map is displayed at a contour level of 1. (δ = 1.0). Electron density corresponding to the metal binding motif is displayed. The metal binding motif is shown in stick representation and colored according to element.

The (NgoL-CTD) crystals belong to space group P2_1_ with cell dimensions of a = 49.5 Å, b = 62.1 Å, c = 92.1 Å and α = γ = 90°, β = 104.6°. The structure was determined by single-wavelength anomalous diffraction (SAD) method using selenomethionine-labeled NgoL-CTD. An experimental electron density map of good quality was obtained and was used to build the model. The structure has been refined to a resolution of 2.4 Å with a final R_free_ and R_crys_ of 27.3% and 22.7%, respectively and the quality of the final model was confirmed through a composite omit map (Figure 1). The final NgoL-CTD structure includes two monomers. For monomer A, residues 463–654 and for monomer B, residues 464–656 could be built and there is no density for five residues extending from 587–591 in both monomers. The structure also shows the presence of 108 water molecules and exhibits good stereochemistry, with 96% of the residues in the favored regions of the Ramachandran plot.

### Structure of the C-terminal domain of NgoL

**Figure 2:**
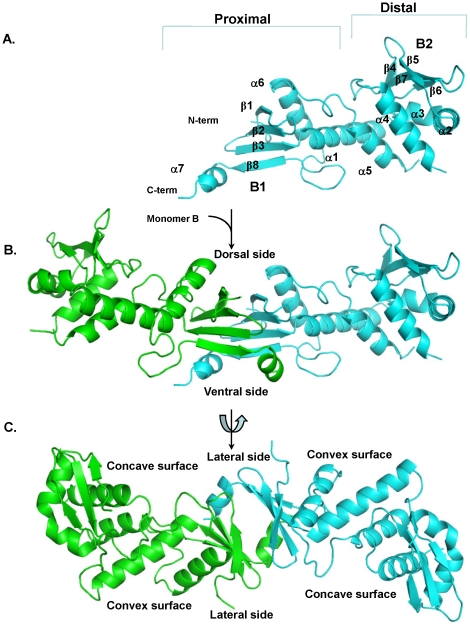
Structure of the NgoL-CTD homodimer. The tertiary and quaternary structure of NgoL-CTD is displayed. (A) The structure of Monomer A (colored cyan) is displayed highlighting the different secondary structural elements. and the two subdomains (B) Side view of the dimer is displayed- Monomer A is colored cyan and Monomer B is colored green. The dorsal and ventral surfaces are labeled. (c) Top view of the NgoL-CTD dimer. The convex and concave surfaces for the two monomers and the two lateral sides of the dimer are highlighted.

The structure of NgoL-CTD shows the presence of an elongated dimer with dimensions 100 Å by 40 Å (Figure 2). Each monomer is bean shaped with concave and convex surfaces (Figure 2A and 2C). In each monomer, towards the N-terminus the polypeptide chain shows the presence of three antiparallel (β1, β2 and β 3) strands of a β-sheet (B1). The polypeptide chain then forms a long alpha helix (α1) that houses the metal binding motif implicated in catalysis. This is followed by a two short strands that are part of smaller four-stranded β -sheet (B2) located at the extremities of the dimer (β4 and β5). After the second strand, the polypeptide chain forms two short helices that are nearly at right angles to each other (α2 and α3). This is followed by two antiparallel strands (β6 and β7), which are part of the four-stranded B2 β-sheet. Next, two helices (α4 and α5) that are roughly of the same lengths are arranged in an anti-parallel manner and the loop between them is disordered (587–591). α5 leads to a nine residue linker that is followed by a helix (α6) which is connected to the fourth strand of the B1 β-sheet (β8) through an ordered 11 residue linker. β8 is followed by a short helix at the C-terminus (α7) that extends towards the other monomer and forms a number of interactions with residues of the second monomer. This C-terminal helix is therefore swapped between the two monomers (Figure 2B). The N-termini of the two monomers and the C-terminus helices are located on the dorsal and ventral sides of the homodimer, respectively. The concave and convex surfaces of the bean-shaped monomers are present on the lateral sides of the dimer. The arrangement of secondary structural elements in each monomer is such that it can be divided into two substructures: (i) a proximal sub-domain composed of the B1 β-sheet, α1, α5, α6 and α7. (ii) distal sub-domain composed of B2 β-sheet, α2, α3 and α4 (Figure 2).

**Figure 3:**
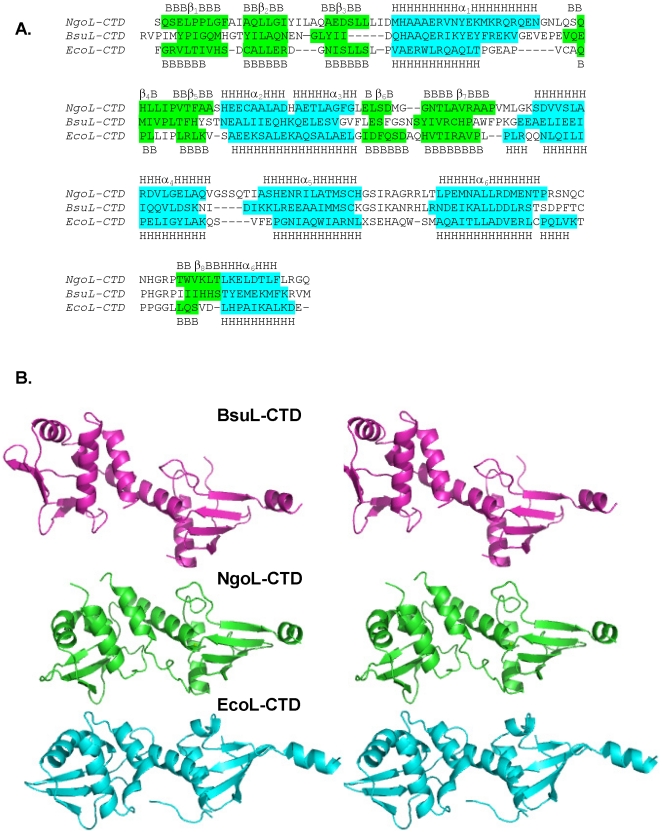
Comparison of the sequence and structures of EcoL-CTD, NgoL-CTD and BsuL-CTD. The sequences and monomer structures of EcoL-CTD, NgoL-CTD and BsuL-CTD were compared. (A) Sequence alignment of the three proteins is displayed and the regions corresponding to β-sheets and α-helices are labeled and shaded green and turquoise, respectively. (B) Stereo figure depicting the structure of monomers of BsuL-CTD (magenta), NgoL-CTD (green) and EcoL-CTD (cyan).

Even though the amino-acid sequence of this protein shows limited similarity with that of the CTD of EcoL, the order and –to an extent- the length of secondary structural elements are similar in the two proteins [17] (Figure 3). Despite this, the two structures cannot be superimposed because the relative spatial orientations of the secondary structural elements are significantly different (Figure 3A and 3B).

In case of BsuL, there is significant sequence similarity (31% identity) with NgoL, and as expected the length and distribution of the secondary elements shows significant overlap (Figure 3). However, in case of BsuL the length of the α7 is shorter and the proximal and distal subdomains exhibit a more stretched and linear arrangement in case of BsuL. Consequently, the concave and convex surfaces that are observed in case in NgoL are absent in case of BsuL. Another major difference between the two structures lies in the orientation of a substructure composed of the B2 sheet and the helices α2 and α3 (residues 514–566). Compared to BsuL, in NgoL, this substructure is rotated through 45° in the counter-clockwise direction in the plane containing the B2 sheet (Figure 3). Since the stretch between β5 and α2 (residues 526–529) is involved in symmetry related interactions, this difference could be due to packing effects. However the observation suggests that this substructure is flexible and can move independently of the rest of the molecule.

### Relation between sequence and structure in MutL homologs

**Figure 4:**
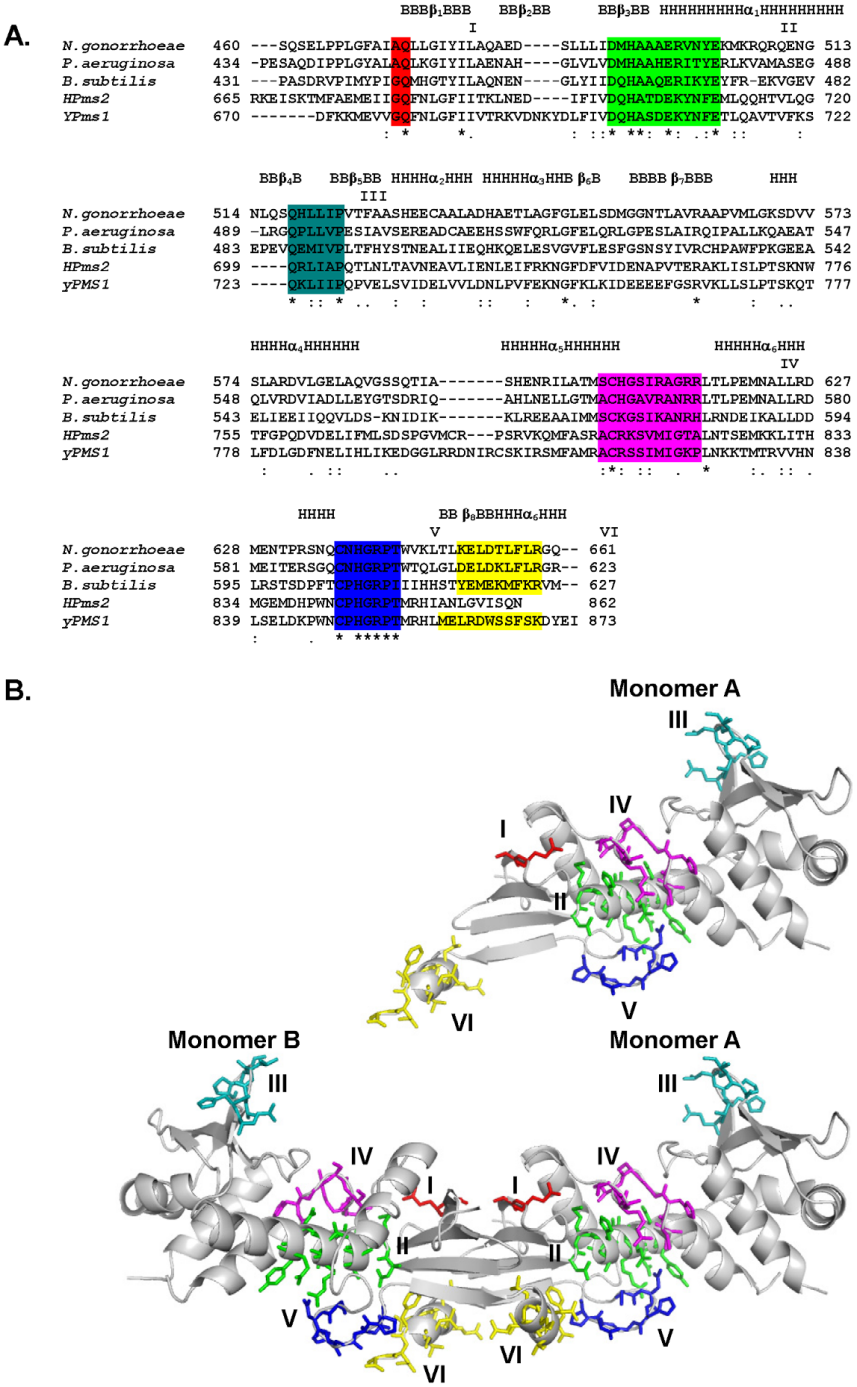
Conserved motifs in the MutL family and the NgoL-CTD structure. The position of the six conserved motifs in the MutL family are displayed in the primary, tertiary and quaternary structures of NgoL-CTD. (A) Sequence alignment of representative sequences corresponding to the CTD regions of homologs of MutL. The stretches corresponding to different secondary structural elements are labeled and the conserved motifs I, II, III, IV, V and VI are shaded red, green, teal, magenta, blue and yellow. (B) The location of the conserved sequence motifs in the NgoL-CTD structure is displayed. The residues of the motifs are displayed in stick representation with the same coloring scheme as before.

On the basis of sequence analysis of the MutL family Kosinski *et al*., had highlighted the presence of six motifs that are conserved to varying degrees [15] (Figure 4A). These consensus sequences map to different regions of the NgoL-CTD structure (Figure 4B). Motif I is highly conserved to a large degree and has the consensus sequence (A/G)Q. It is present in proximal sub-domain (A472-Q473) of NgoL-CTD towards the C-terminus of the β1 strand that is part of the B1 β-sheet involved in dimerization. Motif II has the consensus sequence DQHA(X)_2_E(X)_4_E and represents the metal binding motif (DMHAAAERVNYE in NgoL extending from residues 491 to 502) that is critical for catalysis. In a number of orthologs, including BsuL, the residue following the first Glu residue in this motif is a basic residue (Lys, Arg) and this implies that salt bridge formed between R498 and E502 will be a conserved feature of this motif (equivalent residues in BsuL are R468 and E473).

The consensus sequence for the third motif is QXLLXP, and in NgoL-CTD it is represented by the stretch QHLLIP extending from residues 517–522. This motif is a part of the β4 strand in both NgoL-CTD and BsuL-CTD (QEMIVP) and is located in the distal sub-domain at the extreme ends of the elongated dimer. Among all the six conserved motifs this is the only motif that is present in EcoL-CTD and the location and configuration of this stretch is similar to that seen in NgoL-CTD (Figure 4A). Since, it is located at a distance from the putative active site it is possible that this motif might be involved in mediating protein-protein interactions with MutS or other proteins that interact with MutL to mediate MMR. This motif is also located at the start of the B2+α2+α3 substructure that exhibits a different orientation (with respect to the rest of the dimer) in case of BsuL-CTD. On the basis of homology modeling with β-clamp binding motifs, Pillon et al have suggested that this region might be involved in interactions with the β-clamp although biochemical evidence of a direct interaction between a prokaryotic MutL homolog and β-clamp is still awaited [16].

The fourth motif has the consensus sequence AC(H/R/K)X(A/S)(I/V)(R/K/M)X(G/N) and the corresponding sequence in NgoL-CTD is SCHGSIRAG. Motif IV of NgoL-CTD exhibits better sequence identity with that of prokaryotic MutL homologs. This motif is located in the proximal sub-domain and extends from residues 603–611, and is present between the helices α5 and α6. This stretch contains the basic residues R612 and R613 that are positioned close to the metal-binding site and therefore might be involved in stabilizing DNA. The corresponding stretch in BsuL is SCKGSIKANRH (572–583) and there is no electron density visible for the region GSIKA. The Gln residue present in Motif I also maps close to residues R612 and R613 in NgoL and R582 and H583 in BsuL.

Motif number V has the consensus sequence C(P/N)HGRP, and the NgoL-CTD structure shows that it is located in the proximal sub-domain. This motif is highly conserved and can be considered invariant. In NgoL-CTD, motif V extends from residues 635–640 and is part of an ordered loop located between α6 and β8. Motif V maps close to Motif II with a minimum distance of 4 Å (between side chain of R639 and D491). The HGRP motif is located on one side of a turn-like conformation that is stabilized by a hydrogen bond formed between the backbone atoms of C635 and G638. R639 from this motif forms interactions with residues of the swapped helix α7, and this might add to the stability of the dimer. Due to the observed homology with metal-binding transcription factors, it had been suggested earlier that the two highly conserved motifs II and V are involved in Zn^2+^ binding in the case of hPMS2, but there is no electron density around motif V in case of NgoL-CTD that can be attributed to a bound metal ion [15]. In case of BsuL, residues E468, H464 (Motif II); C573 (Motif IV) and C604 and H606 (Motif V) are involved in stabilizing two Zn^2+^ ions [16]. It should be mentioned here that the substructure harboring this motif in NgoL and BsuL is missing in case of EcoL.

The sixth motif has the consensus sequence (D/E)L(E/D)(K/R)XFxR and the corresponding stretch in NgoL-CTD has the sequence KELDTLFLR, and extends from residues 648–656 (side chain density for the last two residues is not visible). Motif VI of NgoL-CTD exhibits good similarity with that of other prokaryotic MutL homologs. In both NgoL and BsuL, this stretch is part of the C-terminal helix α7 that is swapped between the two monomers.

It is seen that the metal binding motif II is close to all other motifs except III and VI. The number of contacts (within 5 Å) formed by residues of motif II is especially high with residues of motif IV (118). For motif I and V, the number of contacts formed by residues of motif II are 4 and 18, respectively. However, in case of BsuL, motif II forms numerous contacts with motifs IV (65) and V (123) only. It has to be reiterated here that residues of motifs II, IV and V in BsuL come together in space to stabilize two Zn^2+^ ions.

### Metal binding motif

**Figure 5:**
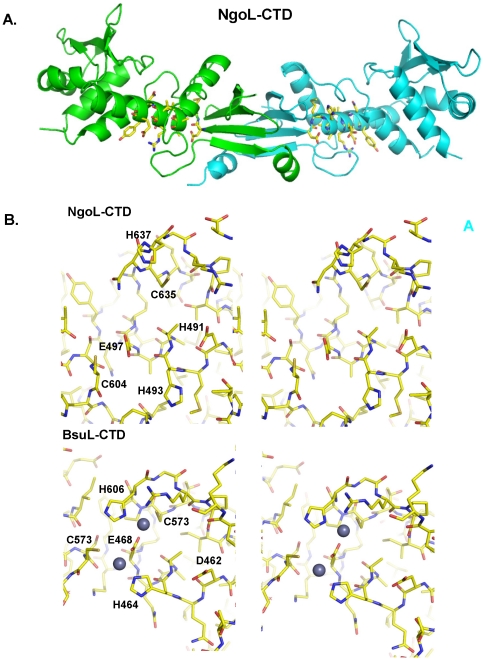
Metal binding motif in NgoL-CTD. The location of the conserved metal binding motif in NgoL-CTD and a comparison with that of BsuL-CTD are displayed. (A) Side view of the NgoL-CTD dimer structure with the metal binding motif displayed in stick representation. (B) Stereo figures displaying a close-up of the metal binding motif in case of NgoL-CTD and BsuL-CTD. Residues are displayed in stick representation and the Zn^2+^ ions in case of BsuL-CTD are displayed as spheres.

The metal binding motif in NgoL-CTD has the sequence DMHAAAERVNYE, which aligns well with the established consensus sequence of DQHA(X)_2_E(X)_4_E, and extends from residues 491 to 502. This region is part of the proximal sub-domain and is located on the lateral sides of the dimer towards the concave surface of each bean-shaped monomer (Figure 5A). The motif folds to adopt a helical configuration and is part of the α1 helix (Figure 2A). The side chains of residues D491, H493, A494, E497, R498, Y501 and E502 are oriented towards the surface of the protein, while the remaining residues are oriented towards the interior of the protein. R498 and E502 interact to form a salt bridge.

In both the monomers, there is residual density present near the residues D491, M492 and H493 and it is ambiguous whether it is due to the presence of a water molecule or a Mg^2+^ ion (Figure S1). There is no octahedral co-ordination visible and the distances from stabilizing groups of protein residues are higher than what has been observed for other complexes. In addition, only two of the six coordinating groups required to stabilize the ion are present at adequate distances, the OD1 atom from the side chain of D491 (2.5 Å) and the backbone carbonyl group from I477 (2.7 Å). Soaking the protein crystals with varying concentrations of Ca^2+^, Sr^2+^ and Ba^2+^ ions did not show any peak in anomalous Fourier maps near this motif and soaking with Mn^2+^ did not yield diffracting crystals. Crystallization experiments with varying concentrations of CaCl_2_, SrCl_2_ or BaCl_2_ in the well solution instead of MgCl_2_ did not yield satisfactory crystals. It is difficult to conclude whether the observed electron density is due to the presence of a water molecule or a divalent Magnesium ion.

**Figure 6:**
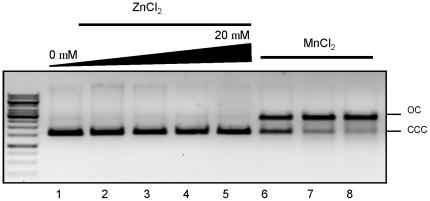
Zn^2+^ ions and endonuclease activity of NgoL-CTD. The effect of increasing metal ion (ZnCl_2_) concentration on endonuclease activity of NgoL-CTD was monitored. It was seen that the enzyme exhibits endonuclease activity in the presence of Mn^2+^ (lanes 6,7 and 8) and not Zn^2+^ ions (lanes 2 to 5).

In case of BsuL-CTD, the metal binding motif is defined by the stretch DQHAAQERIKYEY extending from residues. It has been seen that residues E468 and H464 of the metal binding motif (along with residues of Motif IV and V) are involved in stabilizing Zn^2+^ ions (Figure 5B). In case of BsuL, Zn^2+^ ions do not facilitate endonucleolysis and Mn^2+^ appears to be the primary cofactor ion necessary for activity. In the presence of both Mn^2+^ and Zn^2+^ BsuL exhibits both nicking and double-stranded cleavage activities. NgoL-CTD did not exhibit endonuclease activity (conversion of closed circular form of a plasmid substrate to open circular form) in the presence of Zn^2+^ ions even up to a maximal concentration of 20 mM (Figure 6). Unlike BsuL, NgoL could achieve endonucleolysis in the presence of Mg^2+^ ions also in addition to Mn^2+^ ([13] and Figure 6). Overall, both the enzymes displayed nicking activity in the presence of Mn^2+^ and not Zn^2+^ ions.

### Dimer Arrangement

The described arrangement of the tertiary structure is seen in both the monomers and the two monomers superimpose with an RMSD of 1.3 Å (370 Cα atoms) with the differences in the structure mostly localized to residues that form the dimer interface. In addition, the average B-factor of monomer A is 51.9 Å^2^ whereas that of monomer B is 72.4 Å^2^. The stretches of extending from residues 509–521(β4) and 608–613 (loop between α5 and α6) in monomer B exhibit much higher B-factors than the corresponding regions in A. The higher B-factors imply that these stretches will occupy more than one conformation and therefore can be considered disordered. As expected, the quality of the electron density map in these stretches is much better for monomer A than B. Monomer A is rotated nearly through 180° with respect to monomer B but due to the differences in local structure and B-factors between the two monomers, the two monomers are not related by a crystallographic two fold axis as in case of BsuL.

**Figure 7:**
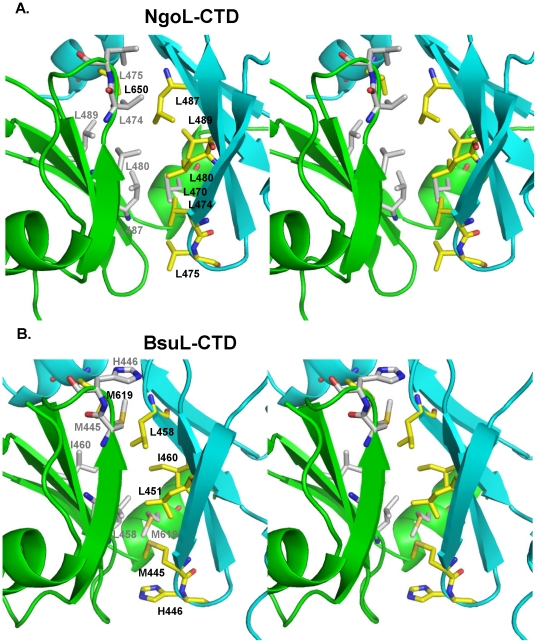
Dimer Interface of NgoL-CTD and BsuL-CTD. The dimer interface lined with hydrophobic residues are displayed in case of NgoL-CTD and BsuL-CTD. Stereo figure displaying the residues that form the hydrophobic surface at the dimer interface in NgoL-CTD (A) and BsuL-CTD (B). The leucines in NgoL-CTD and corresponding residues in BsuL-CTD are shown in stick representation and are colored according to element, with the carbon atoms of leucines from monomer A and monomer B colored yellow and white, respectively.

**Figure 8:**
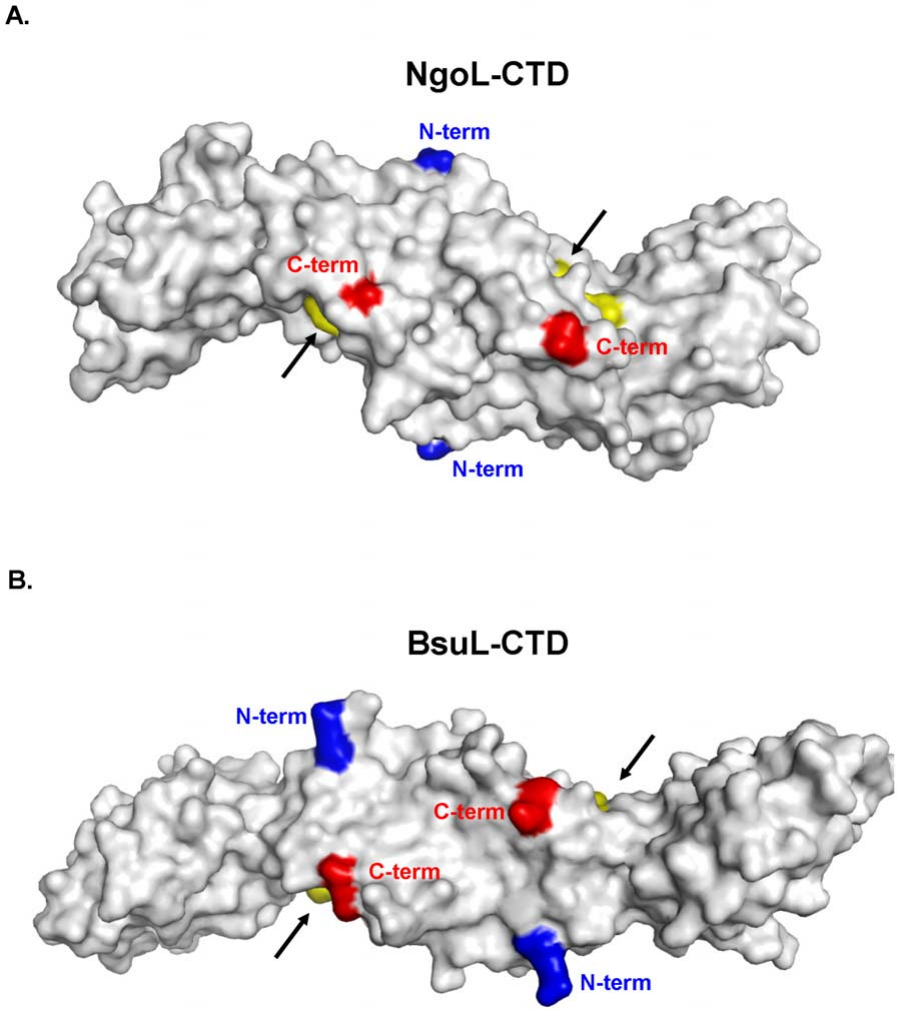
Dimer Arrangement of NgoL-CTD and BsuL-CTD. The location of the putative active site in case of both NgoL-CTD and BsuL-CTD are displayed. The surface of the NgoL-CTD (A) and BsuL-CTD (B) dimers is displayed with key residues of the metal binding motif colored yellow (D491,M492, H493 and E497 for NgoL) and highlighted by arrows. The blue and red patches represent the N- and C- termini, respectively. For clarity, both NgoL-CTD and BsuL-CTD are displayed as viewed from the ventral side. The figure shows that the two metal binding motifs are located on opposite lateral sides of the homodimer.

The dimer interface is primarily formed by juxtaposition of the B1 β-sheet from both monomers. Six leucine residues (L474, L475, L480, L487, L489 and L650) from each monomer are oriented into the interface leading to the formation of highly hydrophobic surfaces that come together to form a very stable dimer (Figure 7A). The presence of these amino acid residues in the interface between the two monomers also imposes a rotational transformation on monomer B to prevent steric interactions between these leucine residues. As a result, the orientation of monomer B is inverted with respect to monomer A (Figure 2C and 8A). Other residues such as F654, I471, Y478 and V643 also contribute to the formation of the hydrophobic patch at the dimer interface. The stability of the homodimer is augmented by polar interactions formed by residues of the α7 helix with residues of the opposite monomer. Thus, the dimer interface is formed from the B1 β-sheet and α7 of the proximal sub-domain from each monomer (Figure 2C and Figure 7A).

In case of BsuL, the residues corresponding to aforementioned six leucines are L451, I460, L458, M445, H446 and M619 (Figure 7B). The identity of these residues is therefore less homogenous than in case of NgoL but the hydrophobic nature of the sidechains is preserved in all but one of them. The buried surface area between the two monomers in NgoL-CTD and BsuL-CTD is 2272 Å^2^ and 2007.4 Å^2^, respectively and this high number provides a quantitative description of the extensive interaction between the two monomers at the dimer interface. In case of NgoL, packing analysis of the contents of the unit cell showed unequivocally that the observed dimer should be the biologically relevant dimer as no other pairing of monomers exhibited a similar degree of interaction in terms of contacts and surface area buried.

The dimeric arrangement of the two monomers in NgoL is such that the concave surfaces housing the putative metal binding sites in each monomer map to the two lateral sides of the protein (Figure 8). Additionally, as the two monomers are inverted with respect to each other the two active sites are located on opposite lateral faces of the elongated dimer (Figure 8A). A similar observation could be made in case of BsuL-CTD. (Figure 8B).

### Mutational Analysis of the NgoL-CTD dimer

**Figure 9:**
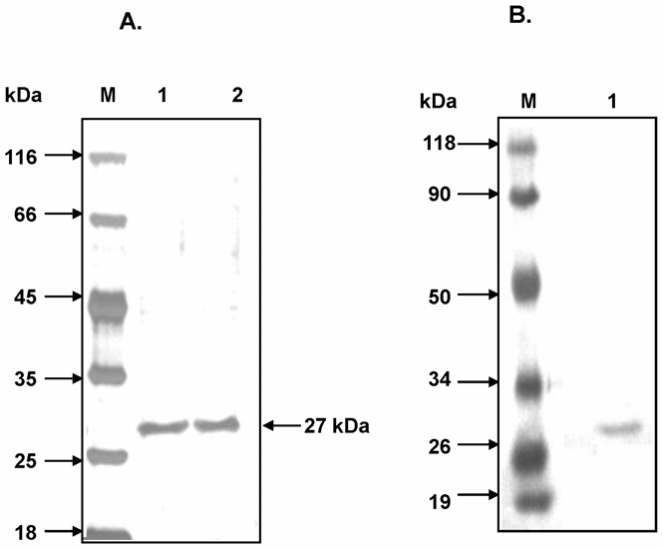
Purity of NgoL-CTD-L480E-L487E. The purity of the mutant protein was assessed. (A). SDS-PAGE analysis of purified NgoL-CTD-L480E-L487E: Lane M, molecular weight markers, lane 1 and 2 purified NgoL-CTD-L480E-L487E. (B). Western blot analysis of NgoL-CTD-L480E-L487E. Lane M molecular weight markers (prestained molecular weight markers), lane 1 NgoL-CTD-L480E-L487E.

The initial structural report of EcoL-CTD reported a dimer arrangement wherein the interface is formed by regions corresponding to the distal sub-domain in NgoL-CTD and this was later corrected [14], [17]. The arrangement of the two dimers seen in case of NgoL-CTD is similar to that deduced by Kosinski *et al*. [14] for MutL-CTD on the basis of rigorous computational analysis and biochemical studies. In order to confirm that the contents of the asymmetric unit represented the true biological dimer, we mutated L480 and L487 located at the dimer interface to Glu. In the dimer interface, L480 from monomer A and L487 from monomer B come close to each other and *vice versa* (Figure 7A). Mutating these residues to Glu would lead to the presence of negatively charged residues within the hydrophobic interface thereby destabilizing the dimer. NgoL-CTD-L480E-L487E was purified to near homogeneity and analyzed, using SDS-PAGE (Figure 9A) and Western blotting (Figure 9B), for alterations in the electrophoretic mobilities and no apparent changes were detected.

**Figure 10:**
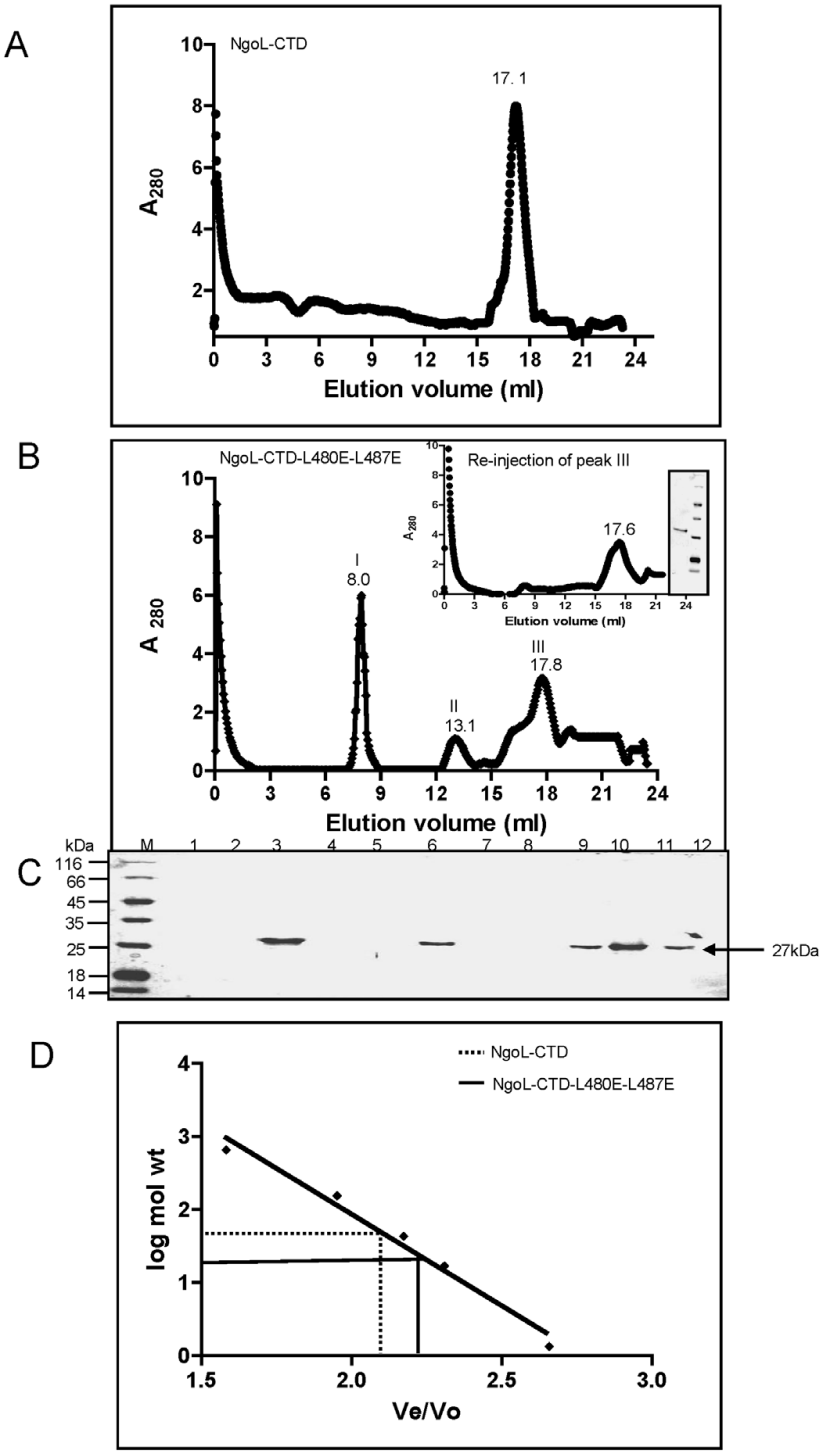
Gel filtration chromatography of the NgoL-CTD wild type and NgoL–CTD-L480E-L487E. The wild-type and mutant protein were both subjected to gel filtration chromatography to determine their oligomeric status. Elution profile of (A) NgoL-CTD wild type and (B) NgoL-CTD-L480E-L487E (C) SDS-PAGE analysis of fractions for (B): Fraction no.'s 3, 6 and 9-11 correspond to peak no.'s I (Rv = 8.0 ml), II (Rv = 13.1 ml) and III (Rv = 17.8 ml), respectively. Peak III was reinjected and the corresponding chromatography and gel analysis profiles are shown as inset figure in (B). (D) The standard curve log molecular weight versus *Ve / Vo* was derived from the elution profiles of the standard molecular weight markers with *Ve* corresponding to the peak elution volume of the protein and *Vo* representing the void volume of the column determined using Blue dextran. The peak positions of wild type NgoL-CTD and NgoL-CTD-L480E-L487E are indicated by dotted and solid lines, respectively.

Gel-filtration analysis was performed to determine size and subunit structure of NgoL-CTD wild type and NgoL-CTD-L480E-L487E mutant in solution. NgoL-CTD wild-type elutes as a dimer of molecular mass 54 kDa (Figure 10A and 10D). This observation is in agreement with the previous report that the protein exists as dimer in solution under native conditions [13]. On the contrary, NgoL-CTD-L480E-L487E eluted as a peak corresponding to molecular mass of 27 kDa (Figure 10B and 10D) which is same as for monomer (peak III). A peak corresponding to molecular mass 660 kDa (Peak II) was also observed. The peak in the void volume (peak I) represents aggregated protein which migrates faster through the matrix because of its non-globular shape. It could be argued that this aggregation was a consequence of primary effect of the mutations leading to the alteration of the structure of the protein. PeakIII was concentrated and reinjected in the gel filtration column and it was observed that majority of the protein again elutes as peak corresponding to molecular mass of 27 kDa (inset, Figure 10B). The gel filtration analysis suggests that a significant fraction of the NgoL-CTD-L480E-L487E protein exists as monomer in solution under native conditions. SDS-PAGE analysis of gel filtration eluted fractions also showed the presence of the protein in peak fractions (Figure 10C). From these results it can be inferred that the amino acid substitution L to E at positions 480 and 487 does alter the oligomeric state and abolishes dimer formation.

**Figure 11:**
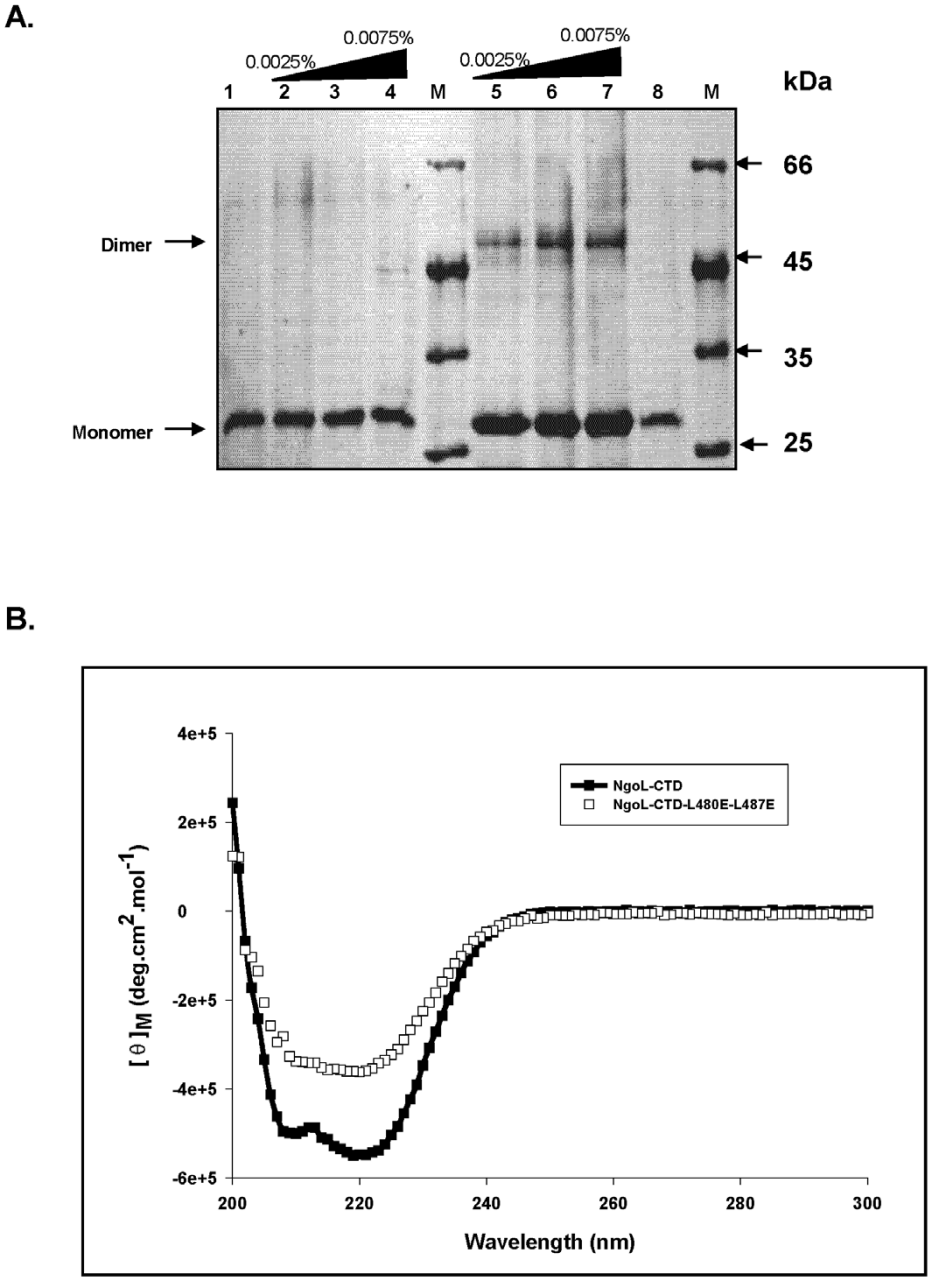
Characterization of NgoL-CTD-L480E-L487E. The change in oligomeric status of the mutant protein was characterized by assessing differences in the products of chemical crosslinking and CD spectra. (A) Chemical crosslinking of NgoL-CTD wild type and NgoL-CTD- L480E-L487E using glutaraldehyde: The NgoL-CTD wild type and NgoL-CTD- L480E-L487E (10 μM) were treated with increasing concentrations of 0.05% glutaraldehyde and products analyzed as described in Materials and methods section. Lane 1, NgoL-CTD-L480E-L487E alone; lane 2, 3 & 4, NgoL-CTD-L480E-L487E and increasing concentration of glutaraldehyde; lane 5, 6 & 7, NgoL-CTD wild type and increasing concentration of glutaraldehyde, lane 8, NgoL-CTD wild type alone; M, molecular weight markers. (B). CD spectra of NgoL-CTD and NgoL-CTD-L480E-L487E. Both the proteins were dialyzed against 10 mM potassium phosphate (pH 8) buffer containing 30 mM KCl. CD spectra were recorded from 200 to 300 nm as described in Materials and methods.

In ordered to get additional information about the oligomeric status, we employed chemical cross-linking of the protein by glutaraldehyde. Glutaraldehyde is a homobifunctional cross-linking reagent that cross-links N-terminal primary amines, resulting in the formation of Schiff's base. Both NgoL-CTD and NgoL-CTD-L480E-L487E were subjected to glutaraldehyde treatment followed by SDS-PAGE analysis. Analysis of the cross-linking results revealed that the wild type formed significant amount of crosslinked product – corresponding to dimer of NgoL-CTD- that increased as a function of glutaraldehyde concentration (Figure 11A). In case of NgoL-CTD-L480E-L487E no crosslinked products were observed and a single band corresponding to a molecular mass of 27 kDa was observed. These results indicate that, the two mutations L480E and L487E completely destabilized the dimer.

Circular dichroism is an excellent method for evaluation of secondary structure, folding and binding properties of a protein. CD spectra were collected from 200–300 nm at 25°C with NgoL-CTD wild type and NgoL-CTD-L480E-L487E protein. Gross conformational changes in the secondary structure were detected between wild type and mutant enzyme (Figure 11B). There is a distinct difference in the magnitude of ellipticity at 208–210 nm between wild type and mutant proteins. A possible explanation is that the helix α7 involved in the dimerization is no longer properly formed in NgoL-CTD-L480E-L487E protein hence leading to a reduced ellipticity at 208–210 nm.

## Discussion

NgoL is the MutL homolog in *Neisseria gonorrhoeae*- a bacterium that is representative of organisms lacking homologs of MutH. In case of these organisms the strategy employed by MMR machinery to discriminate between parental and daughter strand is yet to be determined. Mismatch recognition by MutS is followed by MutL recruitment that is involved in co-ordinating subsequent steps critical for MMR [18]. As in case of humans and yeast, the primary sequence of NgoL reveals the presence of a conserved metal binding motif in the C-terminal domain. The integrity of this metal binding motif has been previously shown to be important for endonucleolytic activity and function in mismatch repair in humans, yeast, *Thermus thermophilus* and *Aquifex aeolicus* [9], [10], [19], [20]. In case of NgoL also, endonucleolytic activity is present in the C-terminal domain that is abrogated by mutations of the invariant residues in the metal binding motif [13].

The structure of NgoL-CTD shows the presence of an elongated dimer which is in congruence with solution studies (Figure 2) [13]. In gel filtration chromatography the protein elutes with a retention volume slightly lower than that of a globular dimer implying a non-spherical shape of NgoL-CTD (Figure 8 and [13]). Even though the sequence of this protein shows limited similarity with that of the CTD of EcoL, the order and extent of secondary structural elements are similar in the two proteins. However, the NgoL structure reveals that the relative spatial orientation of these elements is significantly different between EcoL-CTD and NgoL-CTD and the two structures superimpose with high rmsd. The dissimilar side chains in the two proteins lead to differences in the manner in which the secondary structure elements pack against each other ultimately leading to differences in the tertiary structure. Compared to the bean shaped arrangement in NgoL-CTD, BsuL-CTD appears to attain a more linear and stretched shape. The comparison also suggests that the proximal domain, the distal domain and a substructure in the distal domain (B2+α2+α3) can move independently of each other. It has been suggested before that MutL homologs undergo conformational changes on binding nucleotide and engaging DNA. The observed flexibility in the relative arrangement of sub-domains and sub-structures might facilitate such a mechanism.

As reflected in their B-factors, sections of monomer B appear to be more mobile than corresponding regions in monomer A. Overall, the two monomers in the crystal structure of NgoL-CTD show dissimilarities in structure and a significant difference in mobility of certain stretches and therefore the two monomers are related by a pseudo and not a real twofold axis. The NgoL-CTD homodimer, therefore, exhibits an asymmetric arrangement as compared to EcoL-CTD and BsuL-CTD, where the two monomers are related by a crystallographic two-fold axis [16], [17].

The metal binding motif adopts a helical configuration and is part of the helix α1 (Figure 4). The residual density present near the residues D491, M492 and H493 could be due to the presence of a Mg^2+^ ion. Due to the fact that Mg^2+^ has the same number of electrons as a water molecule, these ions are unambiguously identified from the fact that Mg^2+^ and stabilizing groups arrange together to exhibit a characteristic octahedral geometry. Although structures with bound Mg^2+^ ions exhibiting a co-ordination number less than three have been deposited in the PDB (http://eduliss.bch.ed.ac.uk/tanna/newcngps/mg_cngps_4.htm), the geometry appears to be suboptimal in case of NgoL-CTD. Therefore it is difficult to decide whether this density belongs to a water molecule or a Mg^2+^ ion. It is possible that the density represents a Mg^2+^ ion that exhibits the optimal octahedral geometry only on binding DNA. In case of BsuL, it was seen that residues of Motif II, IV and V were involved in stabilizing two Zn^2+^ ions [16]. However it was seen for both NgoL and BsuL that Zn^2+^ does not support endonucleolysis and Mn^2+^ is probably the primary cofactor ion. Mn^2+^ was found to be important for activity in case of the MutL homologs from humans (PMS2), yeast (PMS1), *Thermus thermophilus* (TthL) and *Aquifex aeolicus* (AaeL). In the presence of both Mn^2+^ and Zn^2+^ ions, BsuL exhibited a double-stranded cleavage activity that would be unwanted in the context of MMR. It is possible that in case of BsuL that Zn^2+^ ions play a structural role and actual catalysis is mediated by Mn^2+^. In case of TthL, it has been seen that if the cysteine residue of Motif V is mutated to Ala, the full length protein still retains activity, but exhibits reduced ATP dependent suppression of endonucleolysis [19].

This helical configuration of the metal binding motif leads to the presentation of the invariant acidic residues in the consensus sequence (D491, E497 and E502) on the same side of the helix towards the surface of the protein. It has been shown previously for NgoL that the double mutant D491N-E497Q exhibits substantial reduction in endonuclease activity [13]. In case of eukaryotic MutLα (MLH1-PMS2) and MutLγ (MLH1-MLH3), mutations in residues corresponding to D491 and E497 have been shown to compromise endonuclease activity and reduce the efficiency of mismatch repair [9], [11], [18], [21]. The importance of E497 can also be ascertained from the fact that a mutation to Lys of the corresponding residue E705 in hPMS2 has been shown to co-segregate with Turcot Syndrome [22]. E497 is located about 8 Å away from the side-chain of D491, and their importance in endonucleolysis implies that the DNA strand straddles these two residues. A similar observation was made from a homology model of the structure of the C-terminal region of the hPMS2:MLH1 heterodimer, and the authors suggested that this implies that more than one metal ion is involved in mediating activity [15]. In case of BsuL-CTD, the residues corresponding to D491 and E497 of NgoL-CTD are D462 and E468. The BsuL-CTD structure shows that E468 is involved in stabilizing both the Zn^2+^ ions. However D462 does not come in contact with the two Zn^2+^ ions, although it has been shown that the D462N mutation abolishes endonuclease activity.

EcoL also shows the presence of a helix in the region corresponding to the metal binding motif in NgoL. This helix is 8 residues (about 2 turns) shorter and lacks the residues necessary to mediate endonucleolytic activity (Figure 3). Thus, by modifying residues located at critical positions in a similar secondary structural arrangement, a catalytic active site capable of creating a nick in DNA is generated in the case of NgoL and BsuL.

In addition to the metal binding motif (II) five other conserved motifs (I, III, IV, V and VI) have previously been described in the MutL family. Out of these, motifs I, IV and V map close to the metal binding motif (II) and all these motifs are present on the proximal sub-domain. From the observed proximity, it can be extrapolated that these motifs (I, II, IV and V) might be important for mediating catalysis by binding substrate DNA and/or co-factor metal ions in the right configuration. Therefore, the active site in case of NgoL-CTD can be considered to be a composite of these four conserved motifs. In case of BsuL-CTD, motif II interacts only with IV and V and there are more contacts observed for V. In this structure, five residues in the centre of motif IV appear to be disordered as the corresponding electron density is not visible. Also, residues of motif V are involved in stabilizing Zn^2+^ along with residues of II. Motif VI is part of the C-terminal helix α7 that is swapped between the two monomers and this implies that helix swapping might be a common feature in homologous proteins. Motif III is located on the distal sub-domain and might be involved in forming protein-protein contacts with other factors involved in MMR such as MutS and the β-clamp. In case of BsuL-CTD, the authors suggest- on the basis of docking studies- that this stretch resembles a β-clamp binding motif and might form interactions with the β-clamp. The (B2+α2+α3) substructure houses motif IV and its observed flexibility might be important in mediating conformational changes critical for function on interaction with partner proteins. It should also be mentioned here that this is the only motif conserved between NgoL-CTD and EcoL-CTD (the degree of similarity is less for BsuL-CTD) and this suggests that interactions mediated through this motif might be conserved between *E. coli* and *N. gonorrhoeae*.

The dimer arrangement seen in the NgoL-CTD crystal structure is similar to that deduced by Kosinski and colleagues for the EcoL-CTD homodimer [14]. Packing analysis of symmetry related molecules showed that no other monomer-monomer pair will show such extensive interactions and lead to this extent of buried surface area. To provide further proof that the contents of the asymmetric unit represent the functional dimer we carried out mutational analysis of the NgoL-CTD dimer interface. The two residues L480 and L487 that are part of the hydrophobic patch present at the interface were mutated to Glu. Characterization of this double mutant clearly showed that the dimer was destabilized leading to the presence of monomeric and aggregated species and a loss of glutaraldehyde mediated crosslinking. Overall, the packing and mutational analysis leaves no doubt that the observed dimer in the Ngol-CTD structure is the functional biological assembly.

On the basis of studies carried out with yeast MutLα, it has been suggested that the dimer arrangement might be different in case of eukaryotes as compared to prokaryotes. Cutalo and colleagues suggest a model wherein the yMutLα heterodimer would be stabilized through interactions formed by a helix in PMS1 that would be the equivalent of α4 in NgoL-CTD [23]. This helix is located in the distal sub-domain far away from the observed dimerization interface and would ultimately lead to a quaternary arrangement that was described in the initial report for EcoL-CTD [17]. However such an arrangement will not provide as extensive an interface as seen in case of NgoL-CTD and will also lack the helix-swapping feature that might be conserved in all MutL homologs. It has been shown previously that nine residues present at the C-terminus of hMLH1 are important for interaction with hPMS2 to stabilize MutLα [24]. Additionally, the mutation L749Q in the C-terminus of hMLH1 has been shown to compromise heterodimerization with hPMS2 [25]. According to a homology-based computational model of human MutLα published earlier, these residues are part of a helix that is swapped between the MLH1 and PMS2 monomers and this helix will correspond to α7 in the NgoL-CTD structure [15]. The dimer arrangement seen in case of CTD of eukaryotic MutLα should therefore be similar to that seen in case of NgoL-CTD and BsuL-CTD and this has been confirmed experimentally recently [26]. It should be mentioned here that – on the basis of sequence analysis- homologs of MutL in thermophilic bacteria do not possess an equivalent of the distal sub-domain in their C-terminal domains [20].

It is expected that the N-terminal domain of NgoL should be located above the CTD with a flexible linker connecting to the two domains. As in case of EcoL, this sort of arrangement will lead to the formation of a large central cavity lined by the NTD dimer on the top, CTD dimer at the bottom and the flexible linker on the sides. For EcoL, it has been suggested that the substrate DNA is looped through the central cavity until the complex of MutL and mismatch bound MutS encounters a MutH molecule bound to a hemi-methylated GATC site [27], [17]. The structure of MutH from *H. influenza* shows that it is a monomeric version of dimeric restriction endonucleases that are known to break phosphodiester bonds on both strands of DNA [28]. The nicking activity of MutH can therefore be explained from the fact that there is only one functional monomer present. In case of heterodimeric MutL homologs found in eukaryotes, only one of the two subunits -PMS2 and not MLH1 in case of humans- harbors the metal binding motif. Thus, nicking activity is achieved by ensuring that only one subunit in the heterodimer has the capacity to break phosphodiester bonds. Therefore, the presence of a similar nicking activity in homodimeric bacterial MutL homologs that harbor the endonuclease activity in the C-terminal domains of both the monomers presents an interesting conundrum.

The inverted dimer, where the two active sites are located on opposite lateral faces of the CTD, provides a potential structural basis for the observed nicking activity of NgoL. Electron microscopy studies have shown that *E. coli* MutS and MutL interact to form DNA loops as a possible mechanism to find a hemi-methylated GATC site in order to achieve accurate strand discrimination [27]. In case of yeast, the MutS homolog (MSH2-MSH3 heterodimer) forms a ternary complex with DNA and MLH1-PMS1 (yMutLα) and this interaction enhances affinity of MutS for the mismatch [29], [30]. It has been suggested that in case of eukaryotic MutLα, DNA is looped through the central cavity of this molecule to detect the signal responsible for strand discrimination [17], [31]. It is possible that in *Neisseria* and other bacteria, a similar mechanism is followed. As has been shown before, this would necessarily involve a close interaction between the MutS and MutL homologs in both prokaryotes and eukaryotes [5], [29], [31], [32]. The presence of active sites on the lateral sides of NgoL-CTD allows for occlusion of one of them due to close apposition of NgoL with MutS or other proteins involved in MMR like the β-clamp. Such a mechanism would ensure that the active site on only one lateral side will be close to the DNA to exert endonucleolytic action and thus avoid the inadvertent formation of extremely deleterious double-stranded breaks.

## Materials and Methods

### Bacterial strains and plasmid vectors

*E. coli* strain DH5*α* (*hsd*R, *rec*A) was used as a host for preparing pUC19. The DNA constructs derived from pET15b were used for overexpression and purification of wild-type NgoL-CTD and NgoL-CTD-L480E-L487E protein. These proteins were expressed in *E. coli* BL21 (DE3) pLysS cells by transforming with appropriate plasmid pET15b constructs.

### Enzymes and chemicals

All reagents used were of analytical or ultra-pure grade. MnCl_2_, BSA, Amp (ampicillin), Hepes, Imidazole and Coomassie Brilliant Blue R-250, proteinase K, phenylmethylsulfonyl fluoride (PMSF), and IPTG were procured from Sigma Chemical Company (USA).

### Purification and Crystallization

The C-terminal domain (residues 458–658) of MutL from *Neisseria* was expressed with a N-terminal His6-tag. Expression and purification was carried out as mentioned before [13]. To prepare selenomethionine (SeMet)–labeled NgoL-CTD, the protein was expressed in B834 strain of *E. coli* auxotrophic for methionine, and the cells were grown using a Se-Met media kit (Molecular Dimensions). The protein crystallized from solutions containing 10–16% PEG 8000 and 0.2 MgCl_2_ buffered at a pH of 8.5. SeMet labeled NgoL-CTD crystallized under similar conditions. Both native and SeMet cocrystals belong to space group P2_1_ with cell dimensions of a = 49 Å, b = 62 Å, c = 92Å and α = γ = 90° and β = 105°. For data collection, the crystals were cryoprotected by soaks for 5 minutes in mother liquor solutions containing 5%, 10%, 15% and 20% glycerol, respectively, and then flash frozen in liquid nitrogen.

### Structure determination

Multiwavelength anomalous diffraction (MAD) data on SeMet crystal (3.3 Å) were measured at the Swiss Light Source (SLS, beamline X06DA). The data was processed using XDS [33]. Although a few Selenium sites could be found with SHELXD, the maps obtained with SHELXE were only partially clear [34]. The quality and cryo-protection of the SeMet crystals was improved considerably and a peak-wavelength SAD dataset of better resolution (2.7 Å) was collected at the European Synchrotron Radiation Facility (ESRF, beamline BM14) along with a native dataset (2.4 Å). All data were indexed and integrated using DENZO and reduced using SCALEPACK (Table 1) [35]. The positions of 9 selenium atoms – out of a possible 16- were found from the SeMet data using the program Shelxc [33]. The program CNS was used for MAD analysis, and also to apply the initial experimental phases (3.0 Å) to Native data and then to extend to 2.4 Å with solvent flattening [36]. This yielded a readily interpretable electron density map which was used to build an initial model of the protein with program O [37]. This was followed by iterative rounds of refinement, model building plus water picking and the *R*_free_ and R_cryst_ converged to final values of 28.6% and 25.2%, respectively. Quality of the chain tracing and side chain conformation in the final model was confirmed using a composite omit map. At this point TLS refinement was carried out using REFMAC (with same test used to calculate R_free_ in CNS) [38]. Proximal sub-domains from each monomer were defined as one rigid unit and the two distal sub-domains were defined as two separate rigid units. TLS refinement lowered the R_free_ and R_cryst_ to 27.3% and 22.8%, respectively (Table 1). The final model of NgoL-CTD includes residues 463–586 and 592–654 (there is no density for eight residues at the N- and C-termini of the construct and for a small loop in the palm domain spanning residues 587–591) and 108 water molecules. The model has excellent stereochemistry, as shown by PROCHECK (CCP4 suite), with about 96% of the residues in the favored regions of the Ramachandran plot [39]. The structure has been deposited in the RCSB with the PDB code 3NCV.

**Table 1:** Data collection and Refinement statistics.

	NgoL-CTD	
Data Collection		
	SeMet	Native
Wavelength	0.97849	0.97871
Space group	P2_1_	P2_1_
Cell dimensions (Å)	49.5 62.6 93.0	49.5 62.1 92.1
(°)	90 106.2 90.0	90.00 104.60 90.00
Resolution (Å)	2.7 Å (2.8-2.7 Å)*	2.4 Å (2.49-2.4 Å)*
*R*_sym_ or *R*_merge_	6.9 (32.9)	4.1 (42.6)
*I*/δ*I*	13.0(2.9)	24.9 (2.1)
Completeness (%)	99.2(98.2)	98.2 (94.2)
Redundancy	6.8 (6.1)	3.0 (2.4)

Refinement		
Resolution (Å)	50-2.40	
No. Reflections	19709	
*R*_work_/*R*_free_	22.7/27.3	
No. atoms		
Protein	2798	
Water	108	
Average B-factors (Å^2^)		
Protein: A	51.9	
Protein: B	72.4	
Water	56.2	
R.m.s deviations		
Bond lengths (Å)	0.0075	
Bond angles (°)	1.56	

*Highest resolution shell is shown in parentheses. *Rmerge  =  Σ|*I* – <*I*>|/Σ*I*, where *I* is the integrated intensity of a given Reflection. †*R*cryst  =  Σ||*F*obs| − |*F*calc||/Σ|*F*obs|. ‡*R*free was calculated using 8% of data excluded from Refinement.

In order to confirm the presence of the metal ion, crystals were soaked with Mn^2+^, Ca^2+^, Ba^2+^ and Sr^2+^ ions in the cryoprotectant solutions and x-ray diffraction data were collected on a Mar345 IP mounted on a Bruker Microstar rotating anode X-ray generator (Molecular Biophysics Unit, Indian Institute of Science). Anomalous Fourier maps were calculated using model phases from the refined structure and did not show the presence of a peak for any of the ions tested. Crystallization experiments were also conducted with MnCl_2_, CaCl_2_, BaCl_2_ and SrCl_2_ in the well solution instead of MgCl_2_ but did not yield diffracting crystals.

### Nicking endonuclease activity in the presence of Zn^2+^

Nicking endonuclease activity was assayed as described previously [13]. Increasing amounts of ZnCl_2_ (0–20 mM) were incubated with 50 nM pUC19 DNA in 10 mM Hepes-KOH (pH 8.0) containing 50 mM KCl, 5 μM NgoL-CTD at 37°c for 60 min and assayed for nicking activity (Figure 6). The control reaction (MnCl_2_) contained 3 μM NgoL-CTD (lane 6) and 5 μM NgoL-CTD(lanes 7 and 8), 50 nM pUC19 DNA in 10 mM Hepes-KOH (pH 8.0) containing 50 mM KCl, 5 mM MnCl_2_ at 37°c for 60 min. The endonuclease activity is measured in terms of increase in quantity of the open circular (OC) form from the closed circular form (CCC) of plasmid substrate. Reactions were stopped with 50 mM EDTA. Proteinase K treatment was given for 30 min at 72°C and the products were analyzed by 1% agarose gel electrophoresis with 90 mM Tris-HCl (pH 8.3), 80 mM boric acid and 2.6 mM EDTA as running buffer.

### Site-directed mutagenesis

Site-directed mutagenesis was performed using PCR-based technique [40] to replace required amino acids. The following oligonucleotides (Sigma, USA) were used for site-directed mutagenesis of NgoL-CTD

(Forward)L480E-L487E: 5′-GGC ATC TAC ATT GAA GCC CAA GCC GAA GAC AGC GAG TTG CTC ATC-3′. (Reverse) L480E-L487E: 5′-CAT GTC GGA CAG TT**C AAG G**CC GAA GCC TGC CAG CGT-3′.

The underlined sequences represent the site of mutation. Sequences in bold are the restriction site lost for that particular mutation. Mutations were introduced into NgoL-CTD gene by using the two-stage megaprimer PCR method. PCRs were carried out with Phusion DNA polymerase (Finnzymes). For each substitution, appropriate forward primer and mutagenic reverse primers were used. In the first round of PCR, oligonucleotide primers mentioned above and pET15b-NgoL-CTD DNA were used to amplify a DNA fragment, which was used as a megaprimer in the second round of PCR. The full-length PCR product was obtained in the second-round PCR by extension of the megaprimer. The PCR product thus obtained was purified, digested with DpnI restriction enzyme to cleave the methylated template DNA, transformed into *E. coli* DH5*α* strain and plated on LB agar containing Amp (100 μg/ml). All mutations were confirmed by DNA sequencing. The resultant plasmids were used for expression and purification of NgoL-CTD-L480E-L487E proteins using the same protocol as before [13].

### Preparation of DNA substrates for nicking assay

Covalently closed circular DNA was used as the substrate to monitor DNA-nicking activity. pUC19 DNA was prepared by transforming *E. coli* DH5*α* cells with pUC19 DNA.

### Molecular size-exclusion chromatography

Gel-filtration experiments were performed using a Superose 6 HR 10/30 column (GE Healthcare). Chromatography was carried out in 10 mM Hepes buffer (pH 8.0) containing 300 mM KCl, 10% glycerol and 1 mM 2-mercaptoethanol. The flow rate was maintained at 0.4 ml/min, and the elution profile was monitored by the absorbance at 280 nm. The void volume (*V*o) was determined using Blue Dextran, and the column was calibrated using the following standard molecular-mass markers (Bio-Rad, USA) Thyroglobulin (670 kDa), **γ**-Globulin (158 kDa), Ovalbumin (44 kDa), Myoglobin (17 kDa), Vitamin B_12_ (1.35 kDa). A standard plot of log (Molecular Mass) versus *Ve/Vo* was prepared and showed the characteristic linear correlation (Figure 10D). The elution volumes (*V*e) of marker proteins and NgoL-CTD wild type and NgoL-CTD-L480E-L487E mutant protein were determined. 0.5 ml fractions were collected and subjected to SDS-PAGE analysis. The molecular masses of proteins were calculated from the standard plot of log (molecular mass) against *V*e/*V*o.

### Chemical Cross-linking

Chemical cross-linking of NgoL-CTD wild type and NgoL-CTD-L480E-L487E protein with glutaraldehyde were carried out by incubating the enzyme (10 μM) on ice for 10 min. Increasing amounts of glutaraldehyde were then added to the protein solution to a final concentration range of 0.0025–0.0075% to the reaction mixture. The reaction mixture was further incubated on ice for 10 min. Reaction was stopped by adding SDS loading dye and boiling the sample for 5 min. Reaction products were separated by electrophoresis and analyzed on a denaturing polyacrylamide gel containing 0.1% (w/v) SDS, 10% (w/v) polyacrylamide. The gel was silver stained to visualize the protein bands.

### CD spectral analysis

CD measurements were recorded on a Jasco J810 polarimeter between 200 and 300 nm in a 2-mm-pathlength quartz cuvette. All experiments were done at 25°C in 10 mM potassium phosphate buffer (pH 8.0) containing 30 mM KCl. The protein solution was incubated for 10 min in a final volume of 400 μl before recording the spectrum. NgoL-CTD and NgoL-CTD-L480E-L487E spectra were recorded at equal concentrations (3 μM). Each experimental spectrum represents the best fit of at least three determinations.

### Miscellaneous methods

For checking the purity, protein samples were separated on 0.1% SDS/10% polyacrylamide gels according to the method described by Laemmli [41]. The protein bands were detected using Coomassie Brilliant Blue R-250. Protein concentration was determined by the method of Bradford [42]. Polyclonal antibodies against the denatured NgoL-CTD protein were raised in rabbits [13] and Western blot analysis was carried out as described in [43]. The structure was analyzed using O and different programs of the CCP4 suite and the figures depicting structure were prepared using Pymol [38], [44].

## Supporting Information

Figure S1 Electron density in the metal binding motif. A close-up of the electron density that could correspond to a water molecule or a Mg^2+^ ion located near the metal binding motif. A simulated annealed Fo-Fc omit map for the H2O/Mg^2+^ is displayed at a contour of 4.0. The residues are displayed in the stick representation and H2O/Mg^2+^ as a sphere.
(2.54 MB TIF)
