# Supplementary figures and images for: The C-Terminal Domain of the MutL Homolog from Neisseria gonorrhoeae Forms an Inverted Homodimer
Source: PLoS One. 2010 Oct 28;5(10):e13726. doi: 10.1371/journal.pone.0013726 (PMC2965676; doi:10.1371/journal.pone.0013726)

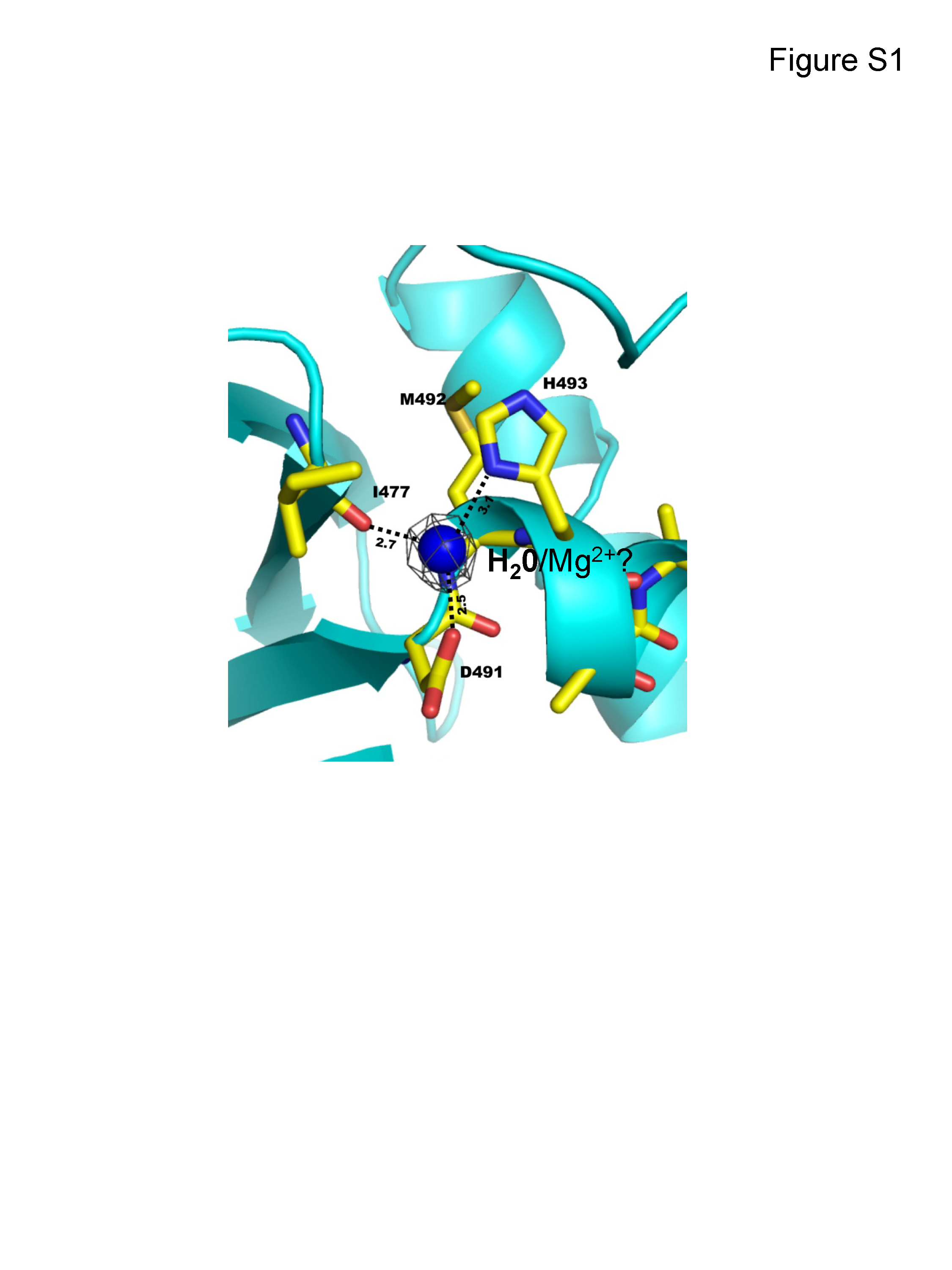

Supplement: Figure S1 — Electron density in the metal binding motif. A close-up of the electron density that could correspond to a water molecule or a Mg2+ ion located near the metal binding motif. A simulated annealed Fo-Fc omit map for the H2O/Mg2+ is displayed at a contour of 4.0. The residues are displayed in the stick representation and H2O/Mg2+ as a sphere. (2.54 MB TIF) [file pone.0013726.s001.tif]
